# Reversal of oral anticoagulation in patients with acute intracerebral hemorrhage

**DOI:** 10.1186/s13054-019-2492-8

**Published:** 2019-06-06

**Authors:** Joji B. Kuramatsu, Jochen A. Sembill, Hagen B. Huttner

**Affiliations:** Department of Neurology, University Hospital Erlangen, Schwabachanlage 6, 91054 Erlangen, Germany

**Keywords:** Intracerebral hemorrhage, Anticoagulation reversal, Tranexamic acid, Ciraparantag, Desmopressin

## Abstract

In light of an aging population with increased cardiovascular comorbidity, the use of oral anticoagulation (OAC) is steadily expanding. A variety of pharmacological alternatives to vitamin K antagonists (VKA) have emerged over recent years (direct oral anticoagulants, DOAC, i.e., dabigatran, rivaroxaban, apixaban, and edoxaban) which show a reduced risk for the occurrence of intracerebral hemorrhage (ICH). Yet, in the event of ICH under OAC (OAC-ICH), hematoma characteristics are similarly severe and clinical outcomes likewise substantially limited in both patients with VKA- and DOAC-ICH, which is why optimal acute hemostatic treatment in all OAC-ICH needs to be guaranteed. Currently, International Guidelines for the hemostatic management of patients with OAC-ICH are updated as several relevant large-sized observational studies and recent trials have established treatment approaches for both VKA- and DOAC-ICH. While the management of VKA-ICH is mainly based on the immediate reversal of elevated levels of international normalized ratio using prothrombin complex concentrates, hemostatic management of DOAC-associated ICH is challenging requiring specific antidotes, notably idarucizumab and andexanet alfa. This review will provide an overview of the latest studies and trials on hemostatic reversal agents and timing and summarizes the effects on hemorrhage progression and clinical outcomes in patients with OAC-ICH.

## Introduction

Of all stroke sub-types, intracerebral hemorrhage (ICH) constitutes roughly 15% and is associated with the worst prognosis [1–3]. Mortality amounts up to 50% after 1 year, and over two thirds of patients survive with significant functional dependency [3, 4]. Over the last years, randomized controlled trials investigating treatment options to influence functional outcome in general ICH populations have failed to provide effective treatment strategies [5–9]. Worldwide, the incidence of intracerebral hemorrhage (ICH) is increasing and primarily driven by low- and middle-income countries [1]. Alongside the demographic change in Western Hemisphere countries, and increasing comorbidity requiring oral anticoagulation (OAC), OAC-associated ICH represents a growing dilemma [10].

Compared to general ICH cohorts, patients with OAC-ICH are older, exhibit larger ICH-volumes, have more frequent intraventricular hemorrhage (IVH), and importantly have a greater frequency of hematoma expansion (HE), all of which are significant outcome predictors determining an even poorer prognosis [11–13]. In vitamin K antagonist (VKA)-associated ICH, the rate of HE is described to occur in more than one out of three patients although international normalized ratio (INR) levels were not elevated beyond the therapeutic range. Further, HE may occur protractedly even exceeding 24 h, if anticoagulation status is not reversed [11–13]. Comparing ICH occurring under use of direct oral anticoagulants (DOAC) versus VKA provides at least similar characteristics regarding validated ICH-specific outcome predictors (neurological status, ICH-volume, IVH, HE rates) and clinical outcomes [14–17]. Hence, aggressive and specific medical management to reverse altered coagulation irrespective of anticoagulant used is essential to reduce HE rates and thereby to potentially influence clinical outcomes [18].

## Vitamin K antagonists

Over the decades, VKA have been the mainstay for anticoagulant treatment in patients with atrial fibrillation (A-fib) [19]. A dose-response relationship between bleeding complications and supra-therapeutic international normalized ratio (INR) levels has been documented with a sharp incidence increase for INR levels above 4 [20]. Nevertheless, ICH occurs commonly at therapeutic INR levels exemplified by the largest available VKA-associated ICH cohort (*n* = 1176) reporting a median INR level of 2.8 with an interquartile range between 2.3 and 3.5 [11]. While DOACs are currently replacing VKA as the primary drug in A-fib-patients, VKA remains the primary anticoagulant for several patient populations with high thromboembolic risk [21, 22]. Current American and International Guidelines for the management of ICH patients do not provide specific recommendations on how to treat VKA-associated ICH in terms of a specific reversal strategy [3, 23]. Particularly, INR values representing full reversal, timing of reversal, and reversal agents to be used are not addressed. However, guidelines are currently being revised as several high-quality publications have substantially added knowledge to the field.

In 2015, a large observational multicenter study (*n* = 1176 patients with VKA-ICH) conducted across 19 tertiary care centers in Germany addressed the questions which INR levels should be achieved to most effectively minimize HE [11]. Results showed (for 853 patients with detailed follow-up imaging) that an INR of less than 1.3 was necessary to reduce the risk of HE (INR < 1.3, HE rate = 27%, versus INR > 1.3, HE rate = 45%). This association was stronger than the earlier achieved and significantly present until 4 h after hospital admission (achieved INR < 1.3 within 4 h, HE rate = 20% versus not achieved HE rate = 42%). Notably, these data resulted from a patient population that arrived roughly at a median of 2 h after symptom onset which is an important factor to be considered as the risk for HE is greatest during the hyper-acute time window (< 3 h), recently verified by a large (*n* = 5435) individual patient data meta-analysis [24]. Therefore, it appears that earlier treatment may translate into a greater effect size for HE prevention. These large-sized investigations strongly support that immediate as well as complete reversal is essential to minimize HE in VKA-ICH, which has also been demonstrated in patients requiring more intense anticoagulation, i.e., mechanical heart valves [22]. In addition to specific reversal treatment, the German multicenter study suggested that lowering of systolic blood pressure below 160 mmHg provided further reductions of HE risk and beyond sole imaging findings significantly reduced in-hospital mortality (odds ratio (OR), 0.6, 95%CI 0.4–0.9) [11]. In this regard, for general ICH populations, a meta-analysis of five randomized controlled trials (*n* = 4360) investigating associations of an intensive blood pressure lowering regime (targeting a systolic blood pressure level < 140 mmHg) versus standard blood pressure lowering (systolic blood pressure level < 180 mmHg) showed promising results for reduced HE rates (OR 0.2; 95% CI 0.7 to 1.0, *p* = 0.06) and for death or dependency rates at 3 months (OR 0.9; 95%CI 0.8 to 1.0, *p* = 0.11). The current AHA guideline for the management of ICH patients recommends to target systolic RR levels of 140 mmHg during the acute phase of ICH [3, 25].

Agents to be used for reversal treatment have been investigated more thoroughly in general populations with VKA-associated major hemorrhages or acute surgical indications. One randomized phase IIIb, multicenter, open-label, non-inferiority trial, in 202 patients with VKA-associated hemorrhage (only 24 ICH-patients), showed that abnormal coagulation was more rapidly reversed by vitamin K and 4-factor PCC (containing coagulation factors II, VII, IX, X) applied using staggered dosing (INR 2–4: 25 IU/kg BW, INR 4–6: 35 IU/kg BW, INR > 6: 50 IU/kg BW) compared to fresh frozen plasma (FFP dosing, INR 2–4: 10 ml/kg, INR 4–6: 12 ml/kg, INR > 6: 15 ml/kg), i.e., INR ≤ 1.3, achieved by PCC + vitamin K in 62.2% versus FFP + vitamin K in 9.6% [26]. In another phase 3 study (*n* = 181), patients with an INR ≥ 2.0 and acute indication for surgery using the same dosing approach were randomized to receive either 4-factor PCC at or FFP each combined with vitamin K. In the PCC group, surgery could be started earlier after infusion in the PPC group (median 3.6 h with interquartile range (IQR) 1.9–10.8) compared to the FFP group (median 8.5 IQR, 2.8–18.7; *p* = 0.01) and the primary outcome of effective hemostasis was achieved more frequently with PCC (PCC 90% versus FF 75%; *p* = 0.01) [27]. Both trials did show no signals regarding an unfavorable safety profile of PCC. In patients with ICH, the randomized controlled INCH trial included patients with VKA-associated ICH and INR levels greater or equal to 2 on hospital admission to compare 4-factor PCC (30 IU/kg BW) versus FFP (20 ml/kg BW) both combined with intravenous vitamin K (10 mg). The primary endpoint was the proportion of patients achieving an INR ≤ 1.2 within 3 h, and the trial was prematurely stopped after enrolment of 54 patients as HE rates significantly differed between treatments [28]. A significantly larger proportion of PCC-treated patients (67%) compared with FFP (9%) reached the target INR of ≤ 1.2 after 3 h (adj. odds ratio 30.6, 95% CI 4.7–197.9; *p* = 0.0003). After 24 h, patients in the FFP treatment group had greater increase in ICH volume (absolute ICH volume difference 16.4 mL, 95% CI 2.9–29.9, *p* = 0.02) and had a fivefold increased risk for HE, defined as a ICH volume increase of greater 33% from initial to follow-up imaging (odds ratio 4.5, 95% CI 1.3–20.4, *p* = 0.02). Although there was a strong trend towards reduced 90-day mortality (PCC, 19% versus FFP, 35%), it did not reach statistical significance likely related to the small sample size [28]. Importantly, reversal in VKA-associated ICH should be accompanied by simultaneous administration of slow (15–30 min) intravenous infusion of 10 mg vitamin K (25 ml normal saline) to restore intrinsic hepatic carboxylation of clotting factors and to achieve prolonged hemostasis [29, 30]. Taken together, there is convincing evidence to recommend immediate reversal in VKA-associated ICH to INR levels < 1.3 as fast as possible and to favor 4-factor PCC over plasma to influence HE rates and clinical outcomes [31].

Management of VKA-associated ICH:

- Immediate INR reversal using 4-factor PCC (25–50 IU/kg BW) and vitamin K (10 mg)
- Targeting complete reversal INR < 1.3 as soon as possible, at least within 4 h
- Timely and serial INR measurements within the acute phase
- Intensive systolic blood pressure reduction, targeting 140 mmHg
- Avoid hypotension, i.e., systolic blood pressure level below 100–120 mmHg

## Direct oral anticoagulants

Currently marketed non-VKA anticoagulants comprise three factor-Xa inhibitors (apixaban, edoxaban, rivaroxaban, inhibition of the conversion of prothrombin to thrombin) and the direct thrombin-inhibitor dabigatran (competitive inhibitor of thrombin, thereby inhibiting fibrin production), all of which are now recommended for primary or secondary stroke prevention in patients with atrial fibrillation over VKA [19]. Compared to VKA these DOACs share similar pharmacokinetic properties such that elimination half-life is short ranging from 6 to 17 h across agents in patients with normal renal function [32]. Therefore, effective OAC may theoretically not be present on admission, but importantly cannot be timely and validly excluded by routine diagnostics [33, 34]. Using conventional coagulation testing does not provide sufficient sensitivity or specificity, and currently, no data is available suggesting a definite threshold for all DOACs below which one can exclude DOAC effect [33]. For a rough qualitative estimate of altered hemostasis in DOAC-treated patients in general, thrombin time (TT), prothrombin time (PT), and/or activated partial thromboplastin time may be used. More specifically, if available, quantitative assessment (time-consuming, roughly 30 min) of dabigatran levels may be achieved with the dilute thrombin time (dTT), ecarin clotting time (ECT), and for apixaban, edoxaban, and rivaroxaban with agent-specific anti-factor Xa levels, further point-of-care testing devices are currently under development or evaluation [33, 34]. Hence, patients with known DOAC intake and ICH should all receive immediate reversal treatment. Timing of last DOAC intake is important as early treatment (2–4 h after ingestion) with active charcoal (50 g), if safely tolerable by the patient, may have the potential to reduce drug absorption [35]. Other more general options may theoretically comprise hemodialysis in dabigatran-related bleedings, but this seems not to be a sensible option in ICH where immediate reversal must be achieved. Currently, DOAC reversal comprises specific and unspecific approaches which will be highlighted in the following.

### Specific reversal antidotes

In DOAC-associated major hemorrhages or patients requiring emergency surgery, several prospective, multicenter, open-label studies investigating the effectiveness of reversal agents are available [36, 37]. Yet, specific analyses of patients with ICH from these studies are not published yet. Reversal agents differ regarding pharmaco- mechanistic and kinetic properties as well as effectiveness across the various DOAC agents. Therefore, at the current stage, there is no evidence regarding the effectiveness of reversal agents to influence HE rates or clinical endpoints in DOAC-associated ICH.

#### Ciraparantag for DOAC-associated ICH

Ciraparantag (syn.: PER977, aripazine), a small molecule (520 Da), was designed to reverse anticoagulatory effect of heparinoids, direct thrombin, and factor-Xa inhibitors and is currently investigated in phase II trials (NCT03172910, NCT03288454). The broad application seems to be an advantage, especially as rapid onset of activity, single-dose application, and long duration of effect have been suggested [38]. A recent phase I/II investigation in 82 healthy male subjects has reported that anticoagulation of edoxaban (60 mg) was reversed within 10–30 min as well as over 24 h by a single-dose ciraparantag (100–300 mg), without increasing procoagulant measures (d-dimer, prothrombin fragments 1.2, and tissue factor pathway inhibitor levels) [39]. Ciraparantag received fast-track designation in 2015; however, currently, it remains uncertain whether or when this agent will be further evaluated in controlled trials targeting FDA approval and market release.

#### Idarucizumab for dabigatran-associated ICH

Specific reversal of dabigatran can be achieved with idarucizumab, which is a non-competitive inhibitor and represents a humanized monoclonal antibody fragment binding to dabigatran with high affinity (350 times greater than thrombin). The formation of this complex between idarucizumab and dabigatran is almost irreversible; hence, anticoagulation persistently reversed, and this complex renally excreted [36, 40]. Idarucizumab (Praxbind®) is administered as two intravenous boluses (2 × 2.5 g) within 15 min and gained approval by the European Medicines Agency (EMA) and Federal Drug Administration (FDA) in 2015 for reversal of dabigatran-associated life-threating bleeding complications or for patients requiring emergency surgery.

The full-cohort analyses of the open-label REVERSE-AD study was published in 2017, comprised overall 503 patients grouped into patients with uncontrolled hemorrhage (group A, *n* = 301) or in need for urgent invasive procedures (group B, *n* = 202) [36]. The primary endpoint consisted of the maximum percentage reversal of the anticoagulant effect, measured by the dTT or ecarin-clotting time within the first 4 h after infusion of idarucizumab. Results provided for the entire cohort that on admission 92% of patients had prolonged bleeding measures and 4 h after reversal treatment the median maximum percentage reversal was 100% [36]. For group A, including 98 patients with intracranial hemorrhage of which 53 patients experienced ICH, the median level of unbound dabigatran was initially 110 ng/ml and after reversal was 20 ng/ml and remained below this level for 24 h suggesting impairment of anticoagulation to be very unlikely. In patients with intracranial hemorrhage, protocolized follow-up imaging was not mandated; therefore, effects on HE rates cannot be reported. In patients with gastrointestinal hemorrhage (*n* = 137), further clinical assessment showed that median time to bleeding cessation was 2.5 h. For the entire cohort, the reported thromboembolic event rate was 5% (24/503) within 30 days and in patients with ICH 6% (3/53) experienced a thromboembolic event, all occurring more than 10 days after idarucizumab administration [36].

Mechanistically, idarucizumab is not expected to generate an intrinsic prothrombotic risk, and the reported events are likely to be associated with the underlying disease. Data specifically in ICH patients is very limited, and smaller case series based on a prospective German nation-wide observational study reported HE rate of 25% (2/8) after reversal with idarucizumab theoretically reflecting a HE rate comparable to VKA-associated ICH patients being reversed to INR levels below 1.3 [41]. Even though randomized data and detailed analysis of ICH patients is lacking, findings of the REVERSE-AD study for dabigatran-associated ICH suggest that the specific antidote idarucizumab provides a rapid, sufficient, and prolonged reversal of anticoagulation effect and should be immediately administered after diagnosis of ICH as two intravenous boluses (2 × 2.5 g) within 15 min [36].

#### Andexanet alfa for factor-Xa-inhibitor-associated ICH

Specific reversal of factor-Xa-inhibitors (rivaroxaban, apixaban) can be accomplished with andexanet alfa (Andexxa®) which has been approved in May 2018 by the FDA. Andexanet alfa has been designed to reverse the anticoagulant activity of both direct and indirect factor-Xa-inhibitors [42]. Andexanet alfa acts as a human decoy receptor binding to the active site of factor-Xa inhibitors with high affinity and possesses no catalytic activity [42]. Therefore, factor-Xa activity is supposedly restored and effect of anticoagulation attenuated. Several studies in healthy subjects have been conducted to evaluate the potential of reversing anticoagulation. More specifically, a two-part randomized controlled phase 3 trial (ANNEXA-A and ANNEXA-R) evaluated effectiveness of andexanet alfa in healthy older volunteers taking either apixaban (ANNEXA-A, *n* = 24) or rivaroxaban (ANNEXA-R, *n* = 27) comparing different dosing (400–960 mg) and application regimes (single bolus and bolus followed by 2-h infusion) [43]. Study results showed that in over 90% anti-Xa activity was reduced during time of treatment with andexanet alfa followed by a rebound after end of infusion [43].

Currently, the phase 4 study (NCT02329327) is ongoing, and most recently, the full study report has been published [37]. For this multicenter, prospective, open-label, single-group study patients with factor-Xa inhibitor (apixaban, edoxaban, rivaroxaban, and enoxaparin) associated hemorrhages within 18 h after the last intake has been published. The coprimary outcomes were the percent change in anti-factor-Xa activity after andexanet treatment and the percentage of patients with excellent or good hemostatic efficacy at 12 h after the end of the infusion, as pre-specified [37]. The treatment protocol comprised a bolus infusion over 15 to 30 min followed by a 2-h infusion, with different dosing dichotomized according to last known intake, i.e., intake within the last 7 h or unknown status received a higher dose of 800 mg over 30 min followed by 960 mg, and last intake > 7 h received 400 mg followed by 480 mg. Included patients (*n* = 352) exhibited dominantly intracranial hemorrhages in 64% (*n* = 227/352), including 241 ICH patients, and 20% with gastrointestinal hemorrhages [37]. By study design efficacy, analysis was conducted for 254 patients and results showed a decrease in anti-factor-Xa activity in over 90% in apixaban and rivaroxaban and in 75% of enoxaparin-treated patients measured 4, 8, and 12 h after infusion. Predefined excellent or good hemostasis assessed 12 h after the end of infusion was achieved in 82% (95% CI 77–87%) of patients.

Specifically, focusing on patients with ICH, study results are not published but have been reported at the International Stroke Conference 2019. In 71 ICH patients eligible for efficacy analyses, HE was reported in 15 patients assessed at 1 h and in 1 patient assessed at 12 h. Therefore, the HE rate can be considered to be at 22% (*n* = 16/71) in ICH patients after andexanet infusion, theoretically again comparable to dabigatran-associated and VKA-associated ICH patients receiving reversal treatment. Interestingly, for the entire cohort, no correlation between hemostatic efficacy and decreased anti-factor-Xa activity was noted, but in ICH patients, a moderate correlation could be shown as area under the curve of 0.64, 95% CI (53–74). A debated matter of concern for this study was the reported thrombotic event rate of 10% (*n* = 34/352) including 4% (*n* = 15) with ischemic stroke and 4% (*n* = 13%) with deep vein thrombosis in light of elevated laboratory surrogates (d-dimer, prothrombin fragments 1 and 2). ANNEXA-4 is still ongoing to further evaluate also patients with edoxaban associated hemorrhages and for more detailed analyses of ICH patients. However, comparing the estimated treatment cost (based on US data) of reversal agents suggests a large discrepancy between idarucizumab (5495 USD) or 4-factor PCC (4000 IU, 5080 USD) and andexanet alfa ranging between 24,000 and 48,000 USD [32, 44]. So far, andexanet alfa is only approved in the USA. The European Medicines Agency (EMA) has agreed to consider andexanet alfa (Ondexxya®) for fast track approval provided results of the ongoing trial. Outside the USA, andexanet alfa can (theoretically already now) be purchased through international pharmacies at extremely expensive costs.

In summary, for factor-Xa inhibitor-related ICH, through providing fast and sufficient effect on hemostasis, andexanet alfa harbors several limitations which make administration more complex as compared to idarucizumab. The hemostatic rebound, the need for continuous infusion, the reported prothrombotic complications, and the financial aspects bare the risk that andexanet alfa will ultimately not be used as frequently as necessary. In addition, similar to idarucizumab, data on HE rates and clinical outcomes are necessary in order to verify clinical relevance of andexanet alfa in ICH patients [36, 37].

### Unspecific reversal approaches

#### PCC for factor-Xa inhibitor-associated ICH

Three categories of human plasma compounds—prothrombin complex concentrates (PCC)—are currently available to restore altered coagulation, i.e., 3-factor PCC (II, IX, X), 4-factor PCC (II, VII, IX, X), and activated PCC (activated VII, II, IX, X, FEIBA) [34]. Experimental data and mostly phase I randomized data have suggested that PCC may have potential to reverse anticoagulation induced by factor-Xa inhibitors [45–48]. For direct comparisons of these factor concentrates, most data is present for anticoagulation treatment with rivaroxaban and edoxaban suggesting effects of 4-factor PCC over 3-factor PCC and FEIBA being comparable if not superior to 4-factor PCC to reverse coagulation. A small cross-over study in 10 healthy volunteers, treated with dabigatran and rivaroxaban, suggested that thrombin generation was improved best by activated 4-factor PCC measured by ex vivo hemostatic testing of PCC derivatives in rivaroxaban-treated but not in dabigatran-treated patients [46]. Direct human in vivo comparisons between FEIBA and 4-factor PCC are not available. Clinically more convincing are data investigated in 35 healthy individuals receiving PCC, comparing 3-factor with 4-factor PCC, which showed for both agents possible reversal properties after rivaroxaban treatment [47]. The largest randomized study was conducted in 110 healthy edoxaban-treated (single dose 60 mg) individuals and compared dosing regimens using 4-factor PCC (50 IU/kg BW, 25 IU/kg BW, 10 IU/kg BW) and determined effect based on bleeding duration and volume after dermal punch biopsy. Results provided that PCC administered only at 50 IU per kg body weight influenced both bleeding endpoints supporting a potential role for in unspecific reversal [48]. No adverse events occurred that were adjudicated to be related to the study medication.

In patients with major hemorrhagic complications and/or ICH under use of rivaroxaban or apixaban, a prospective cohort study (*n* = 84) including 59 patients with intracranial hemorrhage investigated associations of 4-factor PCC with hemostasis rate, as defined per study protocol [49]. For the entire cohort, the median PCC dose was 2000 IU (IQR 1500–2000) or 27 IU/kg BW and “effective” hemostasis was scored in 69% (*n* = 58/84) of patients. For patients with intracranial hemorrhage, ineffective hemostasis was reported in 27% (16/59), conferring to similar HE rates as available for reversal with andexanet or idarucizumab [49]. The thromboembolic rate however was rather low with 4% (3/84). One of the first larger observational studies (*n* = 61) in ICH patients did not show signals that PCC was influencing HE rates (43% *n* = 12/28 received PCC versus 29% *n* = 5/17 without PCC, *p* = 0 .5) [17]. The largest available cohort study (*n* = 190; rivaroxaban, *n* = 142; apixaban, *n* = 26; dabigatran, *n* = 22) was based on the follow-up study (2010–2015) from the German-wide multicenter study (RETRACE-program) and, according to study protocol, included only patients with ICH under known DOAC use [14]. For the differing DOAC agents observed HE rates in patients with detailed follow-up imaging were 33% for rivaroxaban, 48% for apixaban, and 20% for dabigatran, which were not statistically different, but larger sample sizes would be required to determine potential DOAC class associations. Specific analyses of HE rates according to reversal strategies provided that across all agents the median PCC dose given was 2000 IU for rivaroxaban (IQR 1500–2600) and dabigatran (IQR 1650–3000) and 2400 IU for apixaban (IQR 1500–3000). But it has to be recognized that overall less than half of all patients received appropriate dosing (dose ≥ 25 IU/kg BW; 44%, *n* = 65/146) which was recommended during that treatment period.

Current consensus recommendations support higher dosing with 50 IU/kg BW or greater, and within that study, only 5% of patients were treated accordingly; therefore, sensible analyses of this higher-dosed regime was not executable. Upon adjusted analyses, this multicenter study provided no effect of PCC reversal on reduction of HE rates in factor-Xa inhibitor-associated ICH (risk ratio 1.06, 95%CI 0.56–1.98) or on clinical endpoints. However, it is always important to identify patients with high re-bleeding risk in whom aggressive medical treatment possesses greater effect size. For rivaroxaban specific anti-Xa-activity levels of greater of 118 ng/ml were identified to be significantly associated with increased HE risk (level > 118 ng/ml, HE rate 56% versus level ≤ 118 ng/ml, HE rate 17%; *p* = 0.01). Upon further categorizing sub-group analyses, no other association, but systolic blood pressure reduction (< 160 mmHg, risk ratio 0.6, 95% CI (0.36–0.98), *p* = 0.04), could be identified as protective on HE. Therefore, available data suggests that 4-factor PCC at a dose of 50 IU/kg BW may be considered as “second-line” treatment in factor-Xa-inhibitor-associated ICH, if andexanet is unavailable.

#### Other hemostatic agents

Recently, a large randomized trial (*n* = 2325) investigated the effect of the anti-fibrinolytic agent tranexamic acid (1 g Bolus, followed by 1 g infusion over 8 h) on functional outcome after 90 days in patients with primary ICH, but per study protocol excluded patients with OAC [6]. Results provided no significant effect on functional outcome, yet sub-analyses suggested a significant relation with reduced HE (ICH volume > 33%; binary odds ratio 0.8, 95%CI (0.66–0.98), *p* = 0.03). Nevertheless, clinical data on associations of tranexamic acid in OAC-associated hemorrhage are sparse. The large international trial (*n* = 20.211), *Effects of tranexamic acid on death, vascular occlusive events, and blood transfusion in trauma patients with significant haemorrhage (CRASH-2)*, showed a significant risk reduction for bleeding-associated death (relative risk 0.85, 95% CI (0.76–0.96); *p* = 0·008) and did not exclude anticoagulated patients as the study protocol incorporated an uncertainty principle, but OAC use was unlikely in this fairly young (mean age 35 years) study population [50]. Post hoc analyses in traumatic brain injury also provided a decrease of intracranial hemorrhage progression but data on association with OAC are not present [51], but may possibly be generated from the follow-up trial CRASH-3 in traumatic brain injury (NCT01402882). Nevertheless, a smaller multicenter RCT is currently enrolling patients to evaluate tranexamic acid application in DOAC-associated ICH (TICH-NOAC, NCT02866838), yet available experimental data does not support this hypothesis [52]. Following the negative results and safety concerns with an increased rate of thromboembolic complications in the FAST Trial, a phase 3 study on the efficacy of recombinant activated factor VII (rFVIIa) in ICH patients, administration of rFVIIa is currently not recommended [53], yet ex vivo and in vitro studies suggest reversal effects of rivaroxaban and apixaban by recombinant FVIIa [54]. Another consideration refers to patients under dual therapy—OAC and concomitant antiplatelet medication—which is present in roughly 10% of patients (*n* = 290/2504) possibly necessitating adjunctive therapies such as platelet transfusions or desmopressin (DDAVP) treatment [55]. For treatment with platelet transfusions, randomized phase 3 trial data (*n* = 190) in antiplatelet-associated ICH suggests a negative association with functional outcome at 3 months (adjusted common odds ratio 2.05, 95% CI (1.18–3.56); *p* = 0.01) and increased severe adverse events (adjusted common odds ratio 1.79, 95% CI (0.98–3.27)) [5]. Meta-analyses for DDAVP treatment (0.4 mcg per kg BW) in patients with platelet dysfunction or with antiplatelet medication support DDAVP use in patients undergoing surgery to reduce bleeding and transfusion requirements [56]. Specifically, in ICH patients, data is very limited but suggests associations with improved platelet activity [30].

Management of DOAC-associated ICH:

- Consider per oral charcoal (50 g), if last intake < 4 h and safe for the patient
- Intensive systolic blood pressure reduction, targeting 140 mmHg
- Avoid hypotension, i.e., systolic blood pressure level below 100–120 mmHg
- Dabigatran-associated ICH, immediate reversal using Idarucizumab (2 × 2.5 g)
- Factor-Xa inhibitor-associated ICH, immediate specific reversal using andexanet alfa (unknown time window or last intake ≤ 7 h, 800 mg over 30 min followed by 960 mg over 2 h, last intake > 7 h, 400 mg over 15 min followed by 480 mg over 2 h)
- Factor-Xa-inhibitor-associated ICH, immediate unspecific reversal using high-dose 4-factor PCC or activated PCC (both 50 IU/kg BW)
- Consider serial specific coagulation measurement to monitor reversal (dabigatran, dTT, ECT; factor-Xa inhibitors, agent-specific anti-Xa activity).

## Conclusions and future directions

**Fig. 1:**
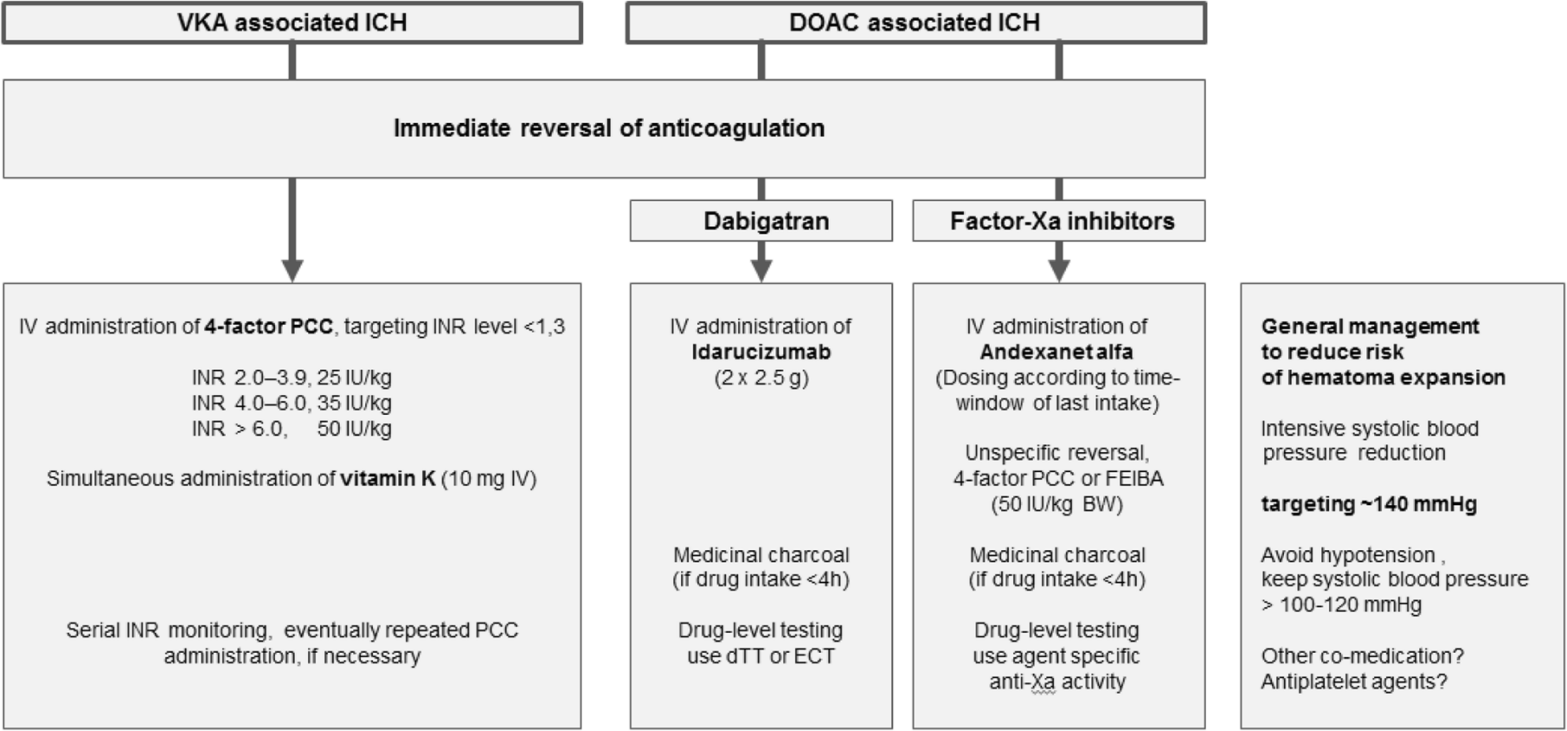
Acute management of anticoagulation-associated intracerebral hemorrhage. Listed values vary according to renal function and drug interactions. Consult product characteristics for individual decision making. h, hours; ICH, intracerebral hemorrhage; IV, intravenous; DOAC, direct oral anticoagulants; PCC, prothrombin complex concentrate; VKA, vitamin K antagonist. FEIBA, activated 4-factor PCC; IU, international units; kg, kilogram; BW, body weight

Significant progress has been achieved recently in large studies for the acute management of patients with OAC-ICH. All these therapeutic interventions mainly focus on reducing the occurrence and extent of hematoma enlargement (please see Fig. 1). On the one hand, blood pressure management targeting systolic levels of 140 mmHg has been verified to limit hematoma expansion and thus should be maintained attentively. On the other hand, optimal hemostatic management significantly restricts hemorrhage progression in all types of OAC-ICH. Specifically, in patients with VKA-ICH, complete reversal of elevated INR levels using prothrombin complex concentrates needs to be initiated immediately to stabilize the intracerebral hematoma. In patients with dabigatran-related ICH, prompt administration of the antidote idarucizumab achieves rapid hemostasis. Although clinical data of minimizing hematoma enlargement by idarucizumab are pending, the likelihood thereof results in a clear recommendation for idarucizumab in dabigatran-ICH. For patients with factor-Xa-inhibitor-associated ICH, andexanet alfa has been demonstrated to provide sufficient hemostasis and first unpublished data suggest effects on reducing hematoma progression in these patients. However, andexanet alfa currently is approved by the FDA only, whereas it is not available elsewhere in the world, and moreover has not been verified for treatment of all factor-Xa-inhibitors. Hence, hemostatic reversal management for factor-Xa-inhibitor-associated ICH remains challenging. Although not sufficiently backed by hemostasiological data, and no formally verified safety analysis, International Guidelines recommend prothrombin complex concentrates administration in dosages of 50 IU/kg bodyweight. Observational analysis on prothrombin complex concentrates administration in factor-Xa-inhibitor-associated ICH showed conflicting data on whether or not there are associations with reduced hemorrhage progression. Two major aspects need to be resolved in timely fashion: firstly, it requires a verification that antidotes, or other prothrombotic drugs respectively, significantly limit hematoma expansion and impact clinical outcomes, and secondly, the all over availability of those drugs needs to be ensured given an increasing worldwide demand.

## Data Availability

Not applicable.

## Abbreviations

BW: Body weight
CI: Confidence interval
DOAC: Direct oral anticoagulants
h: Hours
HE: Hematoma expansion
ICH: Intracerebral hemorrhage
INR: International normalized ratio
IU: International units
kg: Kilogram
OAC: Oral anticoagulation
OAC-ICH: Oral anticoagulation-associated intracerebral hemorrhage
VKA: Vitamin K antagonists
